# The humoral immune response more than one year after SARS-CoV-2 infection: low detection rate of anti-nucleocapsid antibodies via Euroimmun ELISA

**DOI:** 10.1007/s15010-022-01830-x

**Published:** 2022-06-01

**Authors:** Gregor Paul, Philipp Strnad, Oliver Wienand, Ursula Krause, Thomas Plecko, Anja Effenberger-Klein, Katrin Elisabeth Giel, Florian Junne, Annette Galante-Gottschalk, Stefan Ehehalt, Jan Steffen Jürgensen

**Affiliations:** 1 Department of Gastroenterology, Hepatology, Pneumology and Infectious Diseases, Katharinenhospital, Klinikum Stuttgart, Stuttgart, Germany; 2 Department I of Internal Medicine, Division of Infectious Diseases, Faculty of Medicine, University of Cologne, University Hospital Cologne, Cologne, Germany; 3 Department of Nursing Science, Klinikum Stuttgart, Stuttgart, Germany; 4 Service Center, Klinikum Stuttgart, Stuttgart, Germany; 5 Institute of Clinical Chemistry and Laboratory Diagnostics, Klinikum Stuttgart, Stuttgart, Germany; 6 Department of Psychosomatic Medicine and Psychotherapy, Medical University Hospital Tuebingen, Tuebingen, Germany; 7 Department of Psychosomatic Medicine and Psychotherapy, University Hospital Magdeburg, Otto Von Guericke University, Magdeburg, Germany; 8 Public Health Department of Stuttgart, Stuttgart, Germany; 9 Medical Director and Chief Executive Officer, Klinikum Stuttgart, Stuttgart, Germany

**Keywords:** COVID-19, Seroprevalence, Nucleocapsid, Spike, Antibody

## Abstract

**Purpose:**

Antibody assays against SARS-CoV-2 are used in sero-epidemiological studies to estimate the proportion of a population with past infection. IgG antibodies against the spike protein (S-IgG) allow no distinction between infection and vaccination. We evaluated the role of anti-nucleocapsid-IgG (N-IgG) to identify individuals with infection more than one year past infection.

**Methods:**

S- and N-IgG were determined using the Euroimmun enzyme-linked immunosorbent assay (ELISA) in two groups: a randomly selected sample from the population of Stuttgart, Germany, and individuals with PCR-proven SARS-CoV-2 infection. Participants were five years or older. Demographics and comorbidities were registered from participants above 17 years.

**Results:**

Between June 15, 2021 and July 14, 2021, 454 individuals from the random sample participated, as well as 217 individuals with past SARS-CoV-2 infection. Mean time from positive PCR test result to antibody testing was 458.7 days (standard deviation 14.6 days) in the past infection group. In unvaccinated individuals, the seroconversion rate for S-IgG was 25.5% in the random sample and 75% in the past infection group (*P* = < 0.001). In vaccinated individuals, the mean signal ratios for S-IgG were higher in individuals with prior infection (6.9 vs 11.2; *P* = < 0.001). N-IgG were only detectable in 17.1% of participants with past infection. Predictors for detectable N-IgG were older age, male sex, fever, wheezing and in-hospital treatment for COVID-19 and cardiovascular comorbidities.

**Conclusion:**

N-IgG is not a reliable marker for SARS-CoV-2 infection after more than one year. In future, other diagnostic tests are needed to identify individuals with past natural infection.

**Supplementary Information:**

The online version contains supplementary material available at 10.1007/s15010-022-01830-x.

## Background

Since its first emergence end of 2019, the new coronavirus named severe acute respiratory syndrome coronavirus 2 (SARS-CoV-2) has spread globally and caused several waves of infections around the globe [1]. Coronavirus disease 2019 (COVID-19) is now a vaccine-preventable disease, where up to 90% of severe disease forms can be prevented [2]. Seroprevalence studies are now important to provide information on immunity gaps in different populations. Until large-scale vaccination, mainly antibodies against the spike protein of SARS-CoV-2 were used in sero-epidemiological studies [3]. Since all the currently in North America and Europe available vaccines rely on the viral spike protein for induction of neutralizing antibodies, no differentiation between natural infection or vaccination is possible based on anti-SARS-CoV-2-spike-IgG (S-IgG) antibodies only.

Antibodies against viral structures that are not part as an antigen in the vaccines might help in this differentiation. For instance, in hepatitis B serological assays, hepatitis B core antibodies appear shortly after infection and persist for life [4]. Since the hepatitis B core protein is not part of the vaccine, the presence of antibodies against it indicates previous or ongoing infection with the hepatitis B virus.

The nucleocapsid protein (NCP) of SARS-CoV-2 is vital for condensation and packaging of the viral genome and is the most abundant viral protein in infected cells [5, 6]. NCP or nucleotides coding for NCP are not contained in the currently available COVID-19 vaccines. In theory, anti-SARS-CoV-2-nucleocapsid-IgG antibodies (N-IgG) can therefore be used to identify individuals with past SARS-CoV-2 infection. Studies have shown that seroconversion for N-IgG occurs in nearly 100% of hospitalized patients one month after infection [7, 8]. Little is known about the durability of these antibody levels, which is crucial to know for future seroprevalence studies. A short duration would lead to underestimation of individuals with past natural infection in sero-epidemiological studies. We used data acquired from a longitudinal seroprevalence study performed in Stuttgart, Germany, to evaluate the performance of N-IgG in detection of individuals with past COVID-19, based on a sample of individuals with PCR-proven SARS-CoV-2 infection more than one year ago. We also tried to identify predictors for N-IgG positivity one year after infection. Moreover, the humoral immune response of S-IgG after natural infection and after vaccination was studied.

## Materials and methods

### Patients

For the initial seroprevalence study, a representative random sample of 5000 inhabitants of the city of Stuttgart was drawn by the Statistisches Landesamt Baden-Württemberg (regional statistical office) of inhabitants aged five years and older (= control group in this study). Besides the random sample, patients with PCR-proven SARS-CoV-2 infection were actively contacted by the local public health department. Letters containing an information sheet, declaration of consent and a questionnaire were sent to potential study participants or their legal representatives. There were three different versions of the information sheets and declaration of consents available for the age groups 5–12 years, 12–18 years, and 18 years and older. No questionnaire was sent to potential participants younger than 18 years. There was the possibility to complete the questionnaire online or to return a paper version back to the study site via mail or in person. All participants gave written informed consent before participation in the study. For individuals younger than 18 years, parents or a legal representative provided consent.

For capillary blood sampling, the participants had the choice to either visit the study site or book an appointment where members of the study team visited them at home. There were three time points of data collection (first May and June 2020, second November and December 2020 and third June and July 2021). In this study, only data from the third time point were used, since this was the only time point where N-IgG antibodies were analyzed. To study the seroconversion rate for N-IgG shortly after infection, sera from 20 patients that had COVID-19 during the second wave in November 2020 were analyzed. One patient was hospitalized and received supplemental oxygen. The others had mild to moderate symptoms.

To study the humoral response after vaccination, an individual was considered fully vaccinated two weeks after they received the second shot of an mRNA vaccine (Comirnaty, BioNTech-Pfizer or Spikevax, Moderna) or the vector-based vaccine Vaxzevria (AstraZeneca). Participants receiving the COVID-19 vaccine Janssen (Johnson & Johnson) were considered fully vaccinated two weeks after the first shot, as only a single shot was recommended at the time of the study period. Participants with a heterologous Vaxzevria and mRNA prime-boost vaccination were also considered fully vaccinated two weeks after the second dose.

### Laboratory analysis

After pricking of one of the fingertips with a lancet, blood was collected using the Multivette 600 blood collection system by Sarstedt (Sarstedt AG and Co. KG, Nümbrecht, Germany). We assessed anti-SARS-CoV-2 nucleocapsid and spike IgG antibodies by enzyme-linked immunosorbent assays (ELISA) using a commercially available test kit (Euroimmun, Lübeck, Germany, EI 2606–9601 G). Samples were processed on a Dynex DS2 ELISA Processing System (Dynex Technologies, Chantilly, VA USA) according to manufacturer instructions. Results are reported semi-quantitatively by the ratio of extinction of the patient sample over the extinction of a calibrator. A signal was considered positive by calculation of a ratio of 1.1 or higher. The manufacturer considers a signal ratio of 0.8 to < 1.1 as borderline, which was considered negative in this study. A surrogate virus neutralization test (sVNT) was performed using a commercially available kit (GenScript, Piscataway, New Jersey, U.S., L00847). Each assay was performed using an adequate positive and negative control, as recommended by the manufacturer. An inhibition rate of 30% or higher was considered positive for the detection of SARS-CoV-2 neutralizing antibodies. The assay has shown to have a specificity of 100% and a sensitivity of 98–98.6% using this cut-off [9]. There has shown to be no cross-reactivity to other coronaviruses other than SARS-CoV-1, which is not circulating anymore in the population.

### Statistical analysis

Continuous data were expressed as mean with standard deviation or as median and interquartile range depending on data distribution. Categorical variables were reported as number (n) and percentage (%). Statistical differences between groups were determined using a Mann–Whitney U test (continuous variables) or Chi-Squared test and Fisher’s exact test (categorical variables). For analysis of continuous variables with more than two groups, Kruskal–Wallis test with Dunn’s post test was applied. Correlation between S-IgG signal ratios and inhibition rates in sVNT was calculated using a Spearman’s correlation test. Reported p values are 2-tailed, with *P* < 0.05 being considered statistically significant. SPSS (SPSS 24, SPSS Inc., Armonk NY, USA) was used for statistical analysis. Visualization of data was performed using GraphPad Prism (Version 9.3.0, GraphPad Software, San Diego, California USA).

## Results

### Patient and clinical characteristics

From 15th of June 2021 to 14th of July 2021, 454 individuals from the randomly chosen sample from the population (= control group) participated, as well as 217 individuals with PCR-proven SARS-CoV-2 infection in the past. Patient characteristics and comorbidities are shown in (Table 1). In the control group, 57% of participants were female and 52.1% in the group with known past infection. Participants in the control group were significantly younger (median 45 years, interquartile range [IQR] 31–59 years) than in the past infection group (median 48 years, IQR 35–59 years; *P* = 0.009). Mean time from positive PCR test result to antibody testing was 458.7 days (standard deviation [SD] 14.6 days) in the past infection group.

**Table 1:** Demographics and comorbidities in individuals from a randomly selected sample and in individuals with PCR-proven SARS-CoV-2 infection in the past

	Random sample *N* = 454 (%)	Past SARS-CoV-2 infection *N* = 217 (%)	Odds ratio (95% CI)	*P* value
Demographics				
Age, y; median (IQR)	45 (31–59)	48 (35–59)	–	** < 0.05**
Female sex	257 (57)	112 (52.1)	0.82 (0.59–1.14)	0.24
	**N** / **N**_**total**_ (**%**)	**N** / **N**_**total**_ (**%**)		
Full vaccination rate	298 / 443 (67.3)	163 / 211 (77.3)	1.65 (1.13–2.41)	**0.01**
Comorbidities				
Respiratory	16 /302 (5.3)	21 /162 (13)	2.66 (1.35–5.26)	** < 0.01**
Smoking	41 /302 (13.6)	12 /162 (5.5)	1.97 (1–3.86)	** < 0.05**
Cardiovascular	42 /302 (13.9)	25 /162 (15.4)	1.13 (0.66–1.93)	0.68
Liver	1 /302 (0.3)	1 /162 (0.6)	1.87 (0.12–30.09)	1
Type 2 diabetes	9 /302 (3)	4 /162 (2.5)	0.82 (0.25–2.72)	1
Thyroid	32 /302 (10.6)	21 /162 (13)	1.26 (0.7–2.26)	0.45
Musculoskeletal	30 /302 (9.9)	16 /162 (9.9)	0.99 (0.52–1.88)	1
Renal	1 /302 (0.3)	3 /162 (1.9)	5.68 (0.59–55.04)	0.13
Cancer	10 /302 (3.3)	4 /162 (2.5)	0.74 (0.23–2.4)	0.78
Immunodeficiency	8 /302 (2.6)	6 /162 (3.7)	1.41 (0.48–4.15)	0.57
Neurologic	8 /302 (2.6)	4 /162 (2.5)	0.93 (0.28–3.14)	0.59
Psychiatric	9 /302 (3)	7 /162 (4.3)	1.47 (0.54–4.02)	0.44

Bold indicates a *p* value below 0.05

*IQR* interquartile range

Data on comorbidities were available for 302 participants (66.5%) in the control group and 162 (74.6%) in the past infection group, since questionnaires were only sent to individuals 18 years or older. More than half of the participants in both groups had no comorbidities (62.9% in the control group vs. 58.9% in the past infection group). Though, significantly more people in the past infection group had a respiratory comorbidity, such as chronic obstructive pulmonary disease or asthma (5.3 vs. 13%; *P* < 0.01). On the contrary, significantly few people in the past infection group were smokers (13.6 vs. 5.5%; *p* < 0.05). Full vaccination rate was 67.3% in the control group and 77.3% in the past infection group. The difference was statistically significant (*p* < 0.05).

### Seroprevalence rate for anti-SARS-CoV-2-spike-IgG antibodies

**Fig. 1:**
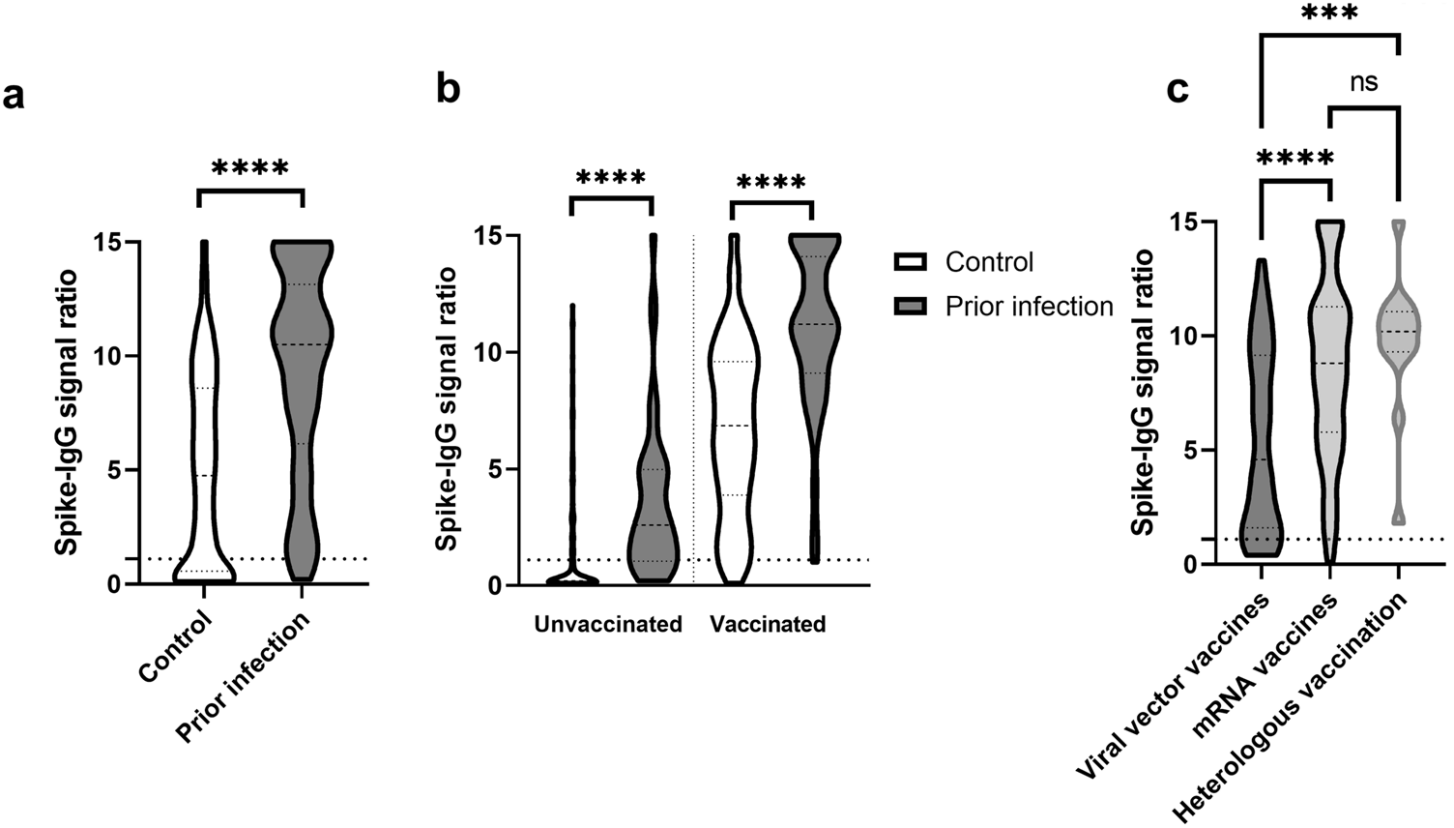
Distribution of anti-SARS-CoV-2-spike antibody ratios between different groups. **a** Violin plot showing the distribution of anti-spike-IgG signal ratios between individuals from a random sample (= control, *N* = 454) and individuals with PCR-proven prior infection (*N* = 217). **b** Shows the effect of vaccination status on the distribution of anti-spike-IgG signal ratios between groups. The groups are comprised of unvaccinated individuals in the control group (*N* = 145), unvaccinated individuals with prior SARS-CoV-2 infection (*N* = 48), vaccinated individuals in the control group (*N* = 298) and vaccinated individuals with prior infection (*N* = 163). **c** Information on the type of vaccination was available from 387 individuals from both groups. The anti-spike-IgG antibody response regarding vaccination with either viral vector vaccines, mRNA vaccines or after heterologous vaccination (viral vector vaccine followed by mRNA vaccine) is shown. The dotted lines represent the signal ratio cutoff of 1.1. A Mann–Whitney *U* test was used for statistical analysis in a and b. Kruskal–Wallis test was used in c. P values less than 0.001 are summarized with three asterisks, and P values less than 0.0001 are summarized with four asterisks. *ns* not significant

In the control group, 71.6% of participants had detectable anti-SARS-CoV-2-Spike-IgG (S-IgG) and 94% in the past infection group (*p* < 0.001). Signal ratios were significantly higher in the past infection group (median 10.5, IQR 6.2–13.1) compared to the control group (median 4.75, IQR 0.6–8.6) (*p* < 0.001) (Fig. 1a). A surrogate viral neutralization (sVNT) assay was performed on a subgroup of participants (*N* = 182 in the random sample group and *N* = 114 in the past infection group) with detectable S-IgG by ELISA (signal ratio ≥ 0.8), to determine the neutralizing activity of the antibodies. In general, there was a good positive correlation between the signal ratios as determined by S-IgG ELISA and inhibition rates in sVNT (*p* < 0.001) (Supplementary Fig. 1). Inhibition rates in sVNT were significantly higher in the past infection group (median 97%, IQR 93–97) compared to the control group (median 87%, IQR 62–96) (*p* < 0.001), indicating higher neutralizing antibody titers.

The seroconversion rate for S-IgG (i.e., signal ratio ≥ 1.1) was 94 and 99.4% for vaccinated individuals in the control and past-infection group, while it was 25.5 and 75% respectively in the unvaccinated group (*p* < 0.001). In vaccinated individuals, the signal ratios for S-IgG were higher after prior infection (median 11.2, IQR 9.15–14.1), when compared to individuals without prior infection (median 6.85, IQR 3.9–9.6; (*p* < 0.001) (Fig. 1b). Vaccinated individuals showed higher inhibition rates of neutralizing antibodies in sVNT after prior infection (median 97%, IQR 96–97), compared to individuals in the control group (median 90%, IQR 65–97) (*p* < 0.001).

Full information on the type of vaccine and the date of last vaccination was available for 387 participants in both groups. 232 (59.9%) were vaccinated with Comirnaty (BioNTech-Pfizer), 64 (16.5%) with Spikevax (Moderna), 74 (19.1%) with Vaxzevria (AstraZeneca), 3 (0.8%) with COVID-19 vaccine Janssen (Johnson & Johnson) and 14 (3.7%) had a heterologous vaccination (viral vector vaccine with Vaxzevria followed by mRNA vaccine). For analysis purposes, the mRNA vaccines Comirnaty and Spikevax were categorized as “mRNA vaccines” and the vaccines Vaxzevria and COVID-19 vaccine Janssen as “viral vector vaccines”. Patients in the viral vector vaccine group were significantly older (median 61 years, IQR 49–65) than patients in the mRNA vaccine group (median 48 years, IQR 35–59; *p* < 0.001). No difference was found in comparison to the heterologous vaccination group (median 51 years, IQR 43–56.75; *p* = 0.44). Time from last vaccination to sample collection was not different between the groups (mRNA median 22.5 days [IQR 10–51.75], viral vector median 27 days [IQR 17–45], heterologous median 39 days [IQR 30.5–47.25], *p* = 0.1). Signal ratios for S-IgG were significantly lower in participants receiving viral vector vaccines compared to mRNA and vaccines and after heterologous vaccination. No difference was seen between mRNA vaccines and heterologous vaccination (Fig. 1c).

On a subgroup of patients, inhibition rates by sVNT, as an indicator for neutralizing antibody titers, were measured (*N* = 51 viral vector vaccines and *N* = 149 for mRNA vaccines). Neutralizing antibody titers were also significantly higher after vaccination with mRNA vaccines (median 96%, IQR 81–97 vs 92%, IQR 63–96, *p* < 0.05). Because of only few participants with heterologous vaccinations, no sVNT was performed in this group.

### Seroprevalence rate for anti-SARS-CoV-2-nucleocapsid-IgG antibodies

In the control group, 4.8% of individuals had detectable N-IgG and 17.1% in the past infection group (*p* < 0.001). Mean signal ratios were significantly higher in the past infection group (mean 0.4, IQR 0.2–0.8) compared to the control group (median 0.1, IQR 0.1–0.2; *p* < 0.001).

Vaccination status had no influence on the seroprevalence of N-IgG antibodies (11.9% in unvaccinated vs. 7.4% in vaccinated individuals; *p* = 0.25). Since N-IgG levels were not followed longitudinally, we wanted to rule out the fact that only a minority of individuals seroconverted shortly after infection, thereby explaining the low detection rate of N-IgG after more than one year. Therefore, N-IgG levels were determined in 20 individuals shortly after COVID-19 (range 14–47 days after positive PCR test result). Seroconversion rate for N-IgG was 100% with a mean signal ratio of 3.54 (range 1.18–7.08).

### Predictors for anti-SARS-CoV-2-nucleocapsid-IgG positivity more than one year after infection

Most of the individuals (82.9%) with PCR-proven SARS-CoV-2 infection more than one year ago had no detectable N-IgG antibodies. To answer the question what predicts N-IgG in the group of patients with known prior infections, we looked at different characteristics, as summarized in Table 2.

**Table 2:** Predictors for detectable anti-SARS-CoV-2-Nucleocapsid-IgG more than one year after infection in patients with PCR-proven SARS-CoV-2 infection

	Anti-SARS-CoV-2-Nucleocapsid-IgG negative *N* = 179	Anti-SARS-CoV-2-Nucleocapsid-IgG positive *N* = 38	Odds ratio (95% CI)	*P* value
Age, years; median (IQR)	46 (50–68)	57 (34–56)	–	** < 0.001**
	**N** / **N**_**total**_ (**%**)	**N** / **N**_**total**_ (**%**)		
Female sex	103 / 179 (57.5)	10 / 38 (26.3)	0.26 (0.12–0.58)	**0.001**
Smoking	11 / 135 (8.1)	1 / 28 (3.6)	2.39 (0.3–19.35)	0.69
In-hospital treatment for COVID-19	9 / 116 (7.8)	6 / 25 (24)	3.75 (1.2–11.77)	< 0.05
Comorbidities				
Respiratory	16 / 134 (11.9)	5 / 28 (17.9)	1.6 (0.54–4.81)	0.37
Liver	0 / 134 (0)	1 / 28 (3.6)	–	0.17
Type 2 diabetes	2 / 134 (1.5)	2 / 28 (7.1)	5.08 (0.68–37.69)	0.14
Musculoskeletal	12 / 134 (9)	4 / 28 (14.3)	1.69 (0.5–5.7)	0.48
Renal	2 / 134 (1.5)	1 / 28 (3.6)	2.44 (0.21–27.93)	0.44
Cardiovascular	14 / 134 (10.4)	11 / 28 (39.3)	5.5 (2.17–14.18)	** < 0.001**
Immunodeficiency	3 / 134 (2.2)	3 / 28 (10.7)	5.24 (1–27.46)	0.07
Cancer	3 / 134 (2.2)	1 / 28 (3.6)	1.62 (0.16–16.1)	0.54
Symptoms				
Fever	67 / 135 (49.6)	22 / 28 (78.6)	3.72 (1.4–9.76)	** < 0.01**
Shivering	33 / 135 (24.4)	9 / 28 (32.1)	1.46 (0.6–3.55)	0.47
Fatigue	94 / 135 (69.6)	22 / 28 (78.6)	1.6 (0.6–4.24)	0.49
Muscle or joint pain	59 / 135 (43.7)	16 / 28 (57.1)	1.7 (0.76–3.91)	0.22
Sore throat	63 / 135 (47)	9 / 28 (32.1)	0.53 (0.23–1.27)	0.21
Cough	86 / 135 (63.7)	21 / 28 (75)	1.71 (0.68–4.31)	0.28
Dyspnea	47 / 135 (34.8)	12 / 28 (42.9)	1.4 (0.61–3.21)	0.52
Wheezing	7 / 135 (5.2)	6 / 28 (21.4)	4.99 (1.53–16.24)	**0.01**
Chest pain	34 / 135 (25.2)	11 / 28 (39.3)	1.92 (0.82–4.51)	0.16
Headache	80 / 135 (59.3)	16 / 28 (57.1)	0.92 (0.4–2.09)	0.84
Nausea	18 / 135 (13.3)	3 / 28 (10.7)	0.78 (0.21–2.85)	1
Abdominal pain	14 / 135 (10.4)	0 / 28 (0)	–	0.13
Coryza	72 / 135 (53.3)	9 / 28 (32.1)	0.41 (0.18–0.98)	0.06
Anosmia	86 / 135 (63.7)	15 / 28 (53.6)	0.66 (0.29–1.5)	0.39

Bold indicates a *p* value below 0.05

*IQR* interquartile range

Individuals with detectable N-IgG antibodies were significantly older (median 57 years, IQR 50–68) in comparison to participants without detectable N-IgG (median 46 years, IQR 34–56; *p* < 0.001). Furthermore, few patients with N-IgG antibodies were female (26.3 vs 57.5%, p < 0.01). Patients with fever above 38 °C (49.6 vs 78.6%; *P* = 0.006) and with wheezing (5.2 vs 21.4%; *P* = 0.01) during their SARS-CoV-2 infection had a higher probability of N-IgG positivity more than one year after infection. The same applies to participants with known cardiovascular comorbidities (10.4 vs 39.3%; *p* < 0.001). Patients that were treated in-hospital due to COVID-19 had a higher seroprevalence rate for N-IgG antibodies (24 vs 7.8% in non-hospitalized; *p* < 0.05).

## Discussion

So far, little is known about the durability of anti-SARS-CoV-2-nucleocapsid-IgG (N-IgG) antibodies more than one year after infection. These antibodies are only detectable after prior infection, while anti-spike-IgG (S-IgG) are also elicited after vaccination.

Here we report new insight into our understanding of the role of N-IgG in detection of individuals with prior SARS-CoV-2 infection. In this study, we show that N-IgG antibodies are only detectable in around 17% of patients around 14 months after PCR-proven infection. Predictors of N-IgG positivity are older age, male sex, cardiovascular comorbidities, fever and wheezing during infection, as well as in-hospital treatment for COVID-19. Furthermore, we show that vaccination leads to the induction of high levels S-IgG antibodies. A single dose after prior infection elicits higher S-IgG titers than two doses without prior infection. Also, mRNA vaccines and heterologous vaccination lead to higher S-IgG antibody titers in comparison to vector-based vaccines.

Data on durability of N-IgG antibodies after COVID-19 are conflicting. The group by Shi et al. looked at the different dynamics of anti-SARS-CoV-2 antibodies over time [7]. After one year, most of the antibodies, including IgA and IgM antibodies against viral spike and nucleocapsid protein dropped below detection rate. Nevertheless, seropositivity rates for N-IgG and S-IgG remained relatively high after one year. Also other groups show relatively stable N-IgG antibody levels up to eight months after infection [10].

On the other hand, Herrington et al. calculate an estimated time to sero-reversion for N-IgG antibodies of 18.6 days for 50% of individuals [11]. Though, they had a large proportion of oligo- or paucisymptomatic patients in their study. As shown in our data and from other groups, disease severity correlates with the height and/or durability of anti-SARS-CoV-2 antibody levels, including N-IgG [8, 12, 13]. Another study performed by van Elslande et al. shows that only 33% of patients with mild COVID-19 were seropositive for N-IgG six months after infection, compared to 69% with severe disease [14]. Besides disease severity as an explanation for differing results, another explanation might be that different serological assays were used. Muecksch et al. showed that in a longitudinal approach, sensitivity of serological assays for anti-SARS-CoV-2 antibodies is dependent on the assay used [15]. Serological assays for antibodies against SARS-CoV-2 often use different epitopes on the same protein, which might influence detection rates [16]. Also, these assays have not been cross-calibrated, making direct comparison between various assays difficult.

Older age is associated with a higher probability for N-IgG positivity more than one year after infection in our study. The mechanism is unknown, but Amjadi et al. show that older age is associated with higher anti-SARS-CoV-2 antibody levels, including N-IgG, after infection [17]. Accordingly, studies have also shown that children have lower N-IgG levels than adults after infection [18]. On the other hand, Van Elslande et al. show in a study performed in COVID-19 patients that gender, like in our study, is associated with N-IgG levels, but not age [19]. On the contrary, they show that age is a significant predictor for S-IgG seropositivity rate, but not gender. The reason for this discrepancy is yet unclear and warrants further research. Older age is the biggest risk factor for severe disease in COVID-19 and the strong inflammatory response in severe disease might drive higher antibody titers [20]. Since higher peak N-IgG antibody levels correlate with a slower decline over time, this might be an explanation for the higher N-IgG positivity rate after 14 months. This has been shown in health care workers as well as hospitalized patients [14, 21]. We were able to show that S-IgG positivity rate in unvaccinated individuals with prior SARS-CoV-2 infection was higher than N-IgG seropositivity rate, suggesting a longer half-live of S-IgG antibodies. The longer duration of S-IgG antibodies was also shown by other groups in healthcare workers and non-severe as well as severe COVID-19 patients, thereby supporting the validity of our data [11, 14, 19]. We show that S-IgG antibody levels were higher in individuals vaccinated once after prior infection, in comparison to a two-dose regimen in individuals naïve to COVID-19. This corroborates the validity of our data since this has been nearly uniformly shown in other studies [22–24]. Additionally, our data show a better humoral antibody response in individuals vaccinated with mRNA vaccines or after heterologous vaccination compared to viral vector vaccines. Neutralization titers are an important predictor of vaccine efficacy in COVID-19 and are in part explaining the superior protection of the currently available mRNA vaccines over viral vector vaccines [25, 26].

Our study has several limitations. First, since N-IgG were only studied once and not longitudinally, we cannot make any conclusions about the trajectory of N-IgG antibodies after infection. Still the quintessence remains that N-IgG antibodies are not a reliable assay to determine past SARS-CoV-2 infection more than one year after infection. Second, another limitation is that in some subgroups, there were only a limited number of events (e.g., comorbidities) reducing the power of the study and increasing the margin of error. This can partially be explained by the fact that questionnaires were only answered by participants 18 years or older, leading to missing data by younger participants. Additionally, filling in the questionnaire was voluntary. The sole motivation for some of the participants was probably the determination of the antibody status. So, some participated in the blood sampling, without answering the questionnaire online or on paper. This is especially true for data about the vaccination status. Though, this does not impair our analysis since we mainly focused on the serological response after vaccination and are putting no spotlight on the level of immunity in the population. Third, in this study, we only use antibody assays from one company (i.e., Euroimmun). Using N-IgG from different manufacturers, which themselves use other epitopes on the NCP or different antibody classes might lead to different results.

A strength of our study is that it describes as one of few studies, the humoral immune response more than one year after SARS-CoV-2 infection. So far, most of the published studies looked at time frames of 12 months or below. We show that nucleocapsid antibodies become undetectable in most patients more than one year after infection. The quickly waning antibody response to NCP, especially in younger individuals with mild infection, would lead to underestimation of past SARS-CoV-2 infection. This has direct implications for the future design of sero-epidemiological studies, where relying on N-IgG antibodies would lead to a false estimation of individuals with prior SARS-CoV-2 infections in the population. Though, the identification of individuals with natural infections has some important implications since we also show that the humoral immune response after vaccination is more pronounced after prior infection. To identify such individuals, where probably vaccines with a lower antigen dose or less doses are sufficient, better assays to identify past natural infection are needed. B-cell epitopes located in the spike and nucleocapsid protein of the virus have shown to be the most reactive, with the lowest chances of cross-reactivity to other coronaviruses, suggesting that antibodies against these two proteins might still be preferred for future diagnostic tests for the humoral response against SARS-CoV-2 [27].

In conclusion, we demonstrate that anti-nucleocapsid-IgG antibodies are not a reliable marker for prior SARS-CoV-2 infection after more than one year after infection. We show that the anti-spike-IgG antibody response is more durable but allows no distinction between prior infection and vaccination. In future, other diagnostic tests are needed to identify individuals with past natural infection after a longer time period.

## Supplementary Information

Below is the link to the electronic supplementary material.

Supplementary file1 Correlation between Spike-IgG signal ratios and inhibition rate measures by surrogate viral neutralization assay. 296 specimens are shown in this graph. A circle represents unvaccinated individuals, a cross individuals with mRNA vaccines and a triangle individuals with viral vector vaccines. The *y*-axis shows the inhibition rate in percent, measured by a surrogate viral neutralization assay. The dotted line represents the cut-off at 30% inhibition. Values 30% or above are considered positive. The *x*-axis shows the signal ratio for anti-spike-IgG antibodies measured by ELISA. (TIF 332 KB)
