# Supplementary figures and images for: The humoral immune response more than one year after SARS-CoV-2 infection: low detection rate of anti-nucleocapsid antibodies via Euroimmun ELISA
Source: Infection. 2022 Jun 1;51(1):83–90. doi: 10.1007/s15010-022-01830-x (PMC9159036; doi:10.1007/s15010-022-01830-x)

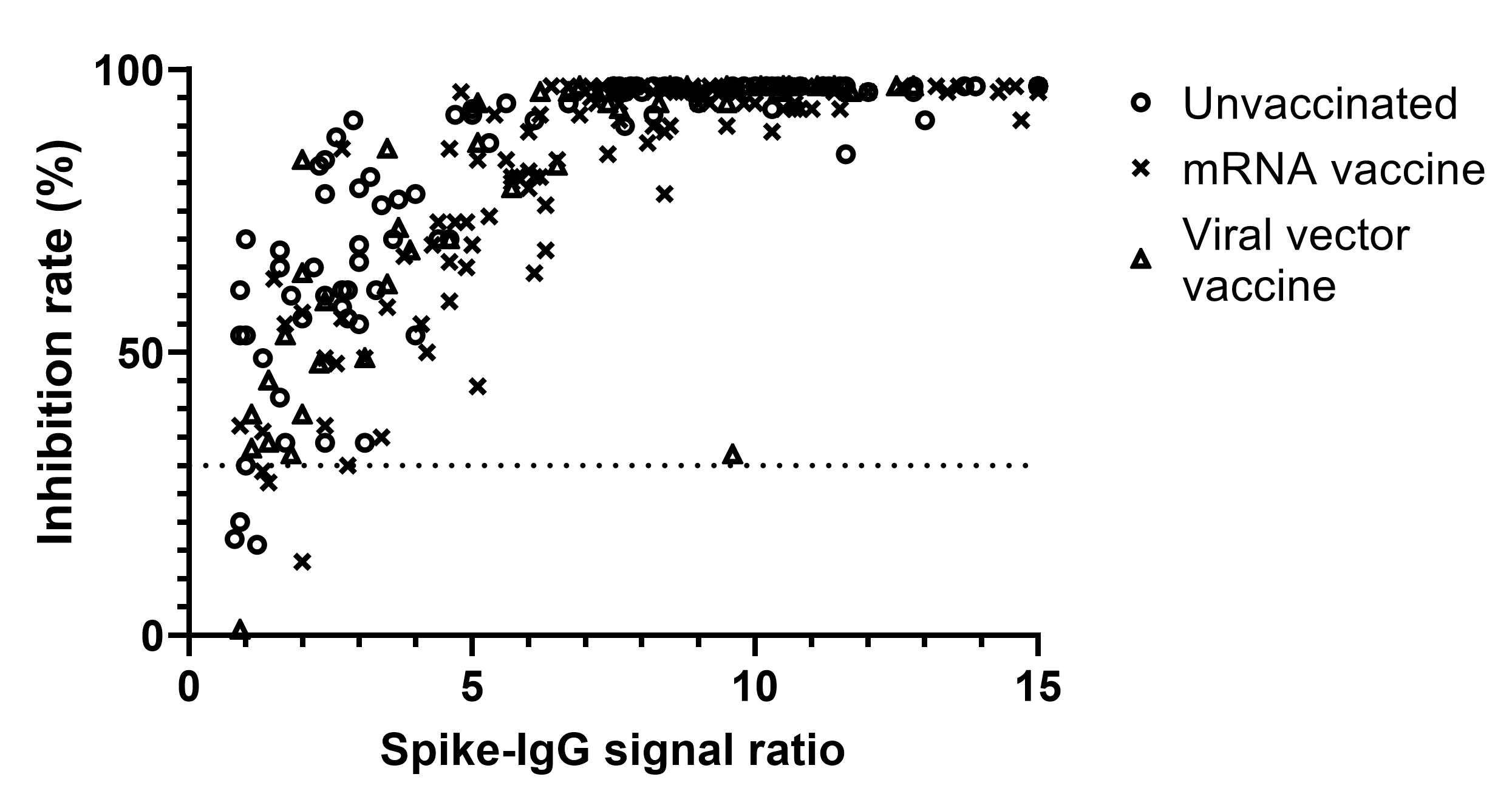

Supplement: Supplementary file 1 — Supplementary file1 Correlation between Spike-IgG signal ratios and inhibition rate measures by surrogate viral neutralization assay. 296 specimens are shown in this graph. A circle represents unvaccinated individuals, a cross individuals with mRNA vaccines and a triangle individuals with viral vector vaccines. The y-axis shows the inhibition rate in percent, measured by a surrogate viral neutralization assay. The dotted line represents the cut-off at 30% inhibition. Values 30% or above are considered positive. The x-axis shows the signal ratio for anti-spike-IgG antibodies measured by ELISA. (TIF 332 KB) [file 15010_2022_1830_MOESM1_ESM.tif]
